# Advances in multiparametric magnetic resonance imaging combined with biomarkers for the diagnosis of high-grade prostate cancer

**DOI:** 10.3389/fsurg.2024.1429831

**Published:** 2024-07-16

**Authors:** Song-lin Li, Ming-yong Zha, Qi Wang, Yong Tang

**Affiliations:** ^1^ Department of Urology, Wuming Hospital of Guangxi Medical University, Nanning, China; ^2^ Department of Experimental Research, Guangxi Medical University Cancer Hospital, Nanning, China; ^3^ Key Laboratory of Early Prevention and Treatment for Regional High-Frequency Tumor (Guangxi Medical University), Ministry of Education, Nanning, Guangxi, China; ^4^ State Key Laboratory of Targeting Oncology, Guangxi Medical University, Nanning, China

**Keywords:** high-grade prostate cancer, multiparametric magnetic resonance imaging, biomarker, pre-diagnosis, Asian

## Abstract

Clinical decisions based on the test results for prostate-specific antigen often result in overdiagnosis and overtreatment. Multiparametric magnetic resonance imaging (mpMRI) can be used to identify high-grade prostate cancer (HGPCa; Gleason score ≥3 + 4); however, certain limitations remain such as inter-reader variability and false negatives. The combination of mpMRI and prostate cancer (PCa) biomarkers (prostate-specific antigen density, Proclarix, *TMPRSS2:ERG* gene fusion, Michigan prostate score, ExoDX prostate intelliscore, four kallikrein score, select molecular diagnosis, prostate health index, and prostate health index density) demonstrates high accuracy in the diagnosis of HGPCa, ensuring that patients avoid unnecessary prostate biopsies with a low leakage rate. This manuscript describes the characteristics and diagnostic performance of each biomarker alone and in combination with mpMRI, with the intension to provide a basis for decision-making in the diagnosis and treatment of HGPCa. Additionally, we explored the applicability of the combination protocol to the Asian population.

## 1 Introduction

Prostate cancer (PCa) is the most common cancer and second leading cause of cancer-related death in males, with a serious effect on their health (1). Prostate-specific antigen (PSA) is the most commonly used screening tool for PCa in the clinic; however, not all patients with PCa have elevated PSA levels, and it is challenging to distinguish PCa from benign diseases, such as prostatitis and benign prostatic hyperplasia (BPH), by relying on PSA ≥ 4 ng/ml alone. Patients with PSA level of 4–10 ng/ml are usually at low-risk for PCa (Gleason score = 6), and the preferred treatment option for patients with low-risk PCa is active surveillance (AS) (2–4). Therefore, clinical decisions based exclusively on PSA are likely to subject patients to unnecessary prostate biopsies and biopsy-induced complications, such as rectal bleeding, infection, urinary retention, and erectile dysfunction, severely impacting their quality of life (5).

Multiparametric magnetic resonance imaging (mpMRI) includes three sequences of T2-weighted imaging (T2WI), diffusion-weighted imaging (DWI), and dynamic contrast enhancement (DCE). A score in the range of 1–5 is obtained based on the prostate imaging reporting and data system (PI-RADS), with the risk of developing high-grade prostate cancer (HGPCa; Gleason score ≥3 + 4) increasing with the score. To accurately diagnose HGPCa and screen candidates for surgery and radiotherapy, the National Comprehensive Cancer Network (NCCN) guidelines recommend that patients with elevated PSA undergo mpMRI before biopsy (3). Clinicians recommend prostate biopsy for patients with PI-RADS ≥ 4 lesions; patients with PI-RADS ≤ 2 lesions do not need to be biopsied. In contrast, PI-RADS 3 lesions lies in the gray area with mpMRI examination; how to guide the biopsy decision in this group of patients has become a significant challenge in the clinic (6). The inter-reader variability is a limitation of mpMRI, reaching 22.0% false negatives in patients with PI-RADS ≤ 3 lesions (7, 8). Therefore, it is necessary to explore new detection methods or feasible combined programs to compensate for the shortcomings of PSA and mpMRI, thus improving the diagnostic accuracy. Biomarkers with a high sensitivity and specificity, convenient sampling, and easy detection for combined testing is the preferred examination method.

This article reviews the research progress of biomarkers of HGPCa and their combined application with mpMRI, aiming to provide a reference for the accurate diagnosis of HGPCa. Although Asians have a lower incidence of PCa than African Americans and Caucasians, approximately 50.0% of Asian patients with PCa have HGPCa as the pathological outcome (9, 10). Notedly, approximately 80.0%–90.0% of clinical trials on mpMRI combined with biomarkers are conducted in Caucasians (11–13), while fewer are conducted in Asian populations. Therefore, we also explored the effectiveness of the combined programs in the Asian population.

## 2 Biomarkers for the diagnosis of HGPCa

### 2.1 Prostate-specific antigen density (PSAD)

PSAD is a PSA derivative, which is calculated with the following formula: PSAD = serum PSA/prostate volume (PV). The diagnosis of HGPCa using the PSAD is susceptible to the influence of the PV. In the PV intervals defined by <50 cm^3^, 50 ≤ PV ≤ 75 cm^3^, and >75 cm^3^, PSAD is a predictor of HGPCa in the range of small [odds ratio (OR): 2.13, *P* = 0.030] to intermediate (OR: 2.80, *P* = 0.010) PVs at a threshold value of 0.15 [ng/ml]/cm^3^. However, when PV is >75 cm^3^, PSAD is not associated with HGPCa (OR: 0.28, *P* = 0.370) (14). Therefore, the ability of PSAD to recognize HGPCa in patients with a very large PV is limited.

### 2.2 Proclarix

Thrombospondin-1 (THBS1) and cathepsin D (CTSD) are two glycoproteins identified to be associated with prostate carcinogenesis by mass spectrometry-based proteomics (15). Klocker et al. (16) combined THBS1, CTSD, total PSA (tPSA), free PSA (fPSA), and age to develop Proclarix, an *in vitro* diagnostic test for predicting HGPCa. The sensitivity, specificity, and negative predictive value (NPV) of the test for diagnosing HGPCa in patients with a PSA of 4–10 ng/ml and PV of 35–250 cm^3^ were 90.0%, 43.0%, and 95.0%, respectively (16). The diagnostic performance of Proclarix is not affected by the PV. Furthermore, it appears to be a good tool for differentiating between HGPCa and BPH. Clinicians using Proclarix to guide biopsy decisions can avoid unnecessary prostate biopsies in 18.2% of patients and reduce mpMRI exams by 25.4%, with a resulting HGPCa misdiagnosis rate of 2.6% (17).

### 2.3 *TMPRSS2:ERG* gene fusion (T2-E)

Androgen-regulated transmembrane serine protease 2 (*TMPRSS2*) is genetically fused with erythrocyte transformation-specific related genes (*ERG*s), leading to the overexpression of ERG proteins, which promote the occurrence and progression of PCa. Tumor cells that detach into the urine after a digital rectal examination (DRE) can be detected by reverse transcription-quantitative polymerase chain reaction (RT-qPCR) and fluorescence *in situ* hybridization (18). Considering its incidence in Asian patients with PCa is only 27.0% (19), this tool is not suitable for the diagnosis of PCa in Asia.

### 2.4 Michigan prostate score (MiPS)

Tomlins et al. constructed a logistic regression model by combining the RNA copy numbers of blood PSA, post-DRE urinary prostate cancer antigen 3 (PCA3), and T2-E fusion genes. This MiPS model produces a range of scores from 0 to 100, reflecting the likelihood of detecting HGPCa on prostate biopsy. Its ability to diagnose HGPCa was superior to a single metric or a combination of the two metrics in this model (20). Although MiPS demonstrated the ability to monitor disease progression in the clinical trial by Eyrich et al. (21), the results of this single-center, small-sample (*n* = 52) study cannot be generalized to the entire patient population under AS.

### 2.5 ExoDX prostate intelliscore (EPI)

The EPI measures the mRNA copy number of three genes, *ERG*, *PCA3*, and *SPDEF*, in the exosomes of prostate tumor cells released into the urine without DRE. RT-qPCR results in a score range of 0–100 that can be used to infer the likelihood of patients with PSA in the gray region to develop HGPCa (22). EPI had an area under the curve (AUC) of 0.700 and an NPV of 90.1% for diagnosing HGPCa when using 15.6 as a threshold to guide biopsy decisions, thus avoiding unnecessary biopsies in 26.0% of patients (23–25). Based on its NPV and AUC, EPI is more suitable as an exclusionary indicator. The idea that EPI was suitable for excluding patients with biopsy-negative, low-risk PCa was confirmed in a follow-up study by Tutrone's team (26). Over 92.0% of the patients with an EPI < 15.6 were low-risk patients who were much less likely to progress to HGPCa at 2.5 years after the EPI examination compared to patients with an EPI > 15.6 (7.9% vs. 26.8%, *P* < 0.001). The EPI reflects disease progression over long time periods and is, therefore, suitable before biopsy and may be applicable to patients under AS.

### 2.6 Four kallikrein score(4Kscore)

The 4Kscore is a predictive model of HGPCa risk that combines four different kinin-releasing enzymes in the blood: tPSA, fPSA, intact PSA (iPSA), and human kallikrein-related peptidase 2 (hk2), as in addition to three clinical variables (age, DRE, and previous biopsy results) to construct a risk prediction model for HGPCa (27). The 4Kscore showed high accuracy in diagnosing HGPCa in patients before biopsy and those under AS, with AUCs of 0.779 and 0.780, respectively (27, 28).

### 2.7 Select molecular diagnosis (SelectMDx)

SelectMDx is a logistic regression model constructed by detecting mRNA levels of *HOXC6* and *DLX1* gene transcripts in urine samples after DRE, combined with the following clinical factors: age, DRE, PSA, PSAD, and family history. It has an AUC for the diagnosis of HGPCa before the initial biopsy of 0.684 (29), and a concordance index of only 0.670 for predicting progression in patients under AS (30). Therefore, SelectMDx should be explored in combined protocols to accurately diagnose HGPCa and monitor patients under AS.

### 2.8 Prostate health index (PHI)

The PHI measures blood levels of fPSA, tPSA, and [−2] PSA prosoma ([−2] proPSA). The HGPCa prediction PHI score is calculated by the following formula: PHI =[([−2] proPSA/fPSA) × tPSA^1/2^]. The Food and Drug Administration has approved PHI for patients aged >50 years with a PSA level 4–10 ng/ml and negative DRE (31). However, thus, far, there is no consensus on the PHI threshold; the current PHI thresholds used in the literature to diagnose HGPCa range from 27 to 67, corresponding to different sensitivities and specificities (32). In areas with a high incidence of PCa, in high-risk populations, or when mpMRI is not available, it is recommended to use a threshold of 62, which has 89.0% specificity, and to combine it with PSA to avoid unnecessary biopsies or MRI. In areas of lower incidence or in non-high-risk populations, it is recommended to use a threshold of 27, which has 100.0% sensitivity and could help avoid missed diagnoses. Multiple PHI examinations are an effective way to monitor disease progression in patients under AS. The risk of progression to HGPCa in patients with PHI ≥ 36 is 2.12 times higher than in patients with PHI < 27 (hazard ratio (HR) = 2.12, 95% confidence interval (CI): 1.00–4.50, *P* = 0.002) (33).

### 2.9 Prostate health index density (PHID)

Similar to PSAD, the diagnostic performance of PHID is limited by the PV, which is indicated for patients with a PV ≤ 50 cm^3^ (34). In 306 patients with a median PV of 37.9 cm^3^ and PSA 4–10 ng/ml, PHID diagnosed HGPCa with an AUC of up to 0.826, which is superior to PSA, PSAD, and PHI (35). In the group of patients with a PV > 50 cm^3^, PHID demonstrated a diagnostic power similar to PSA (AUC: 0.686 vs. 0.700, respectively) (34).

## 3 MpMRI combined with biomarkers for the diagnosis of HGPCa

### 3.1 MpMRI combined with PSAD

Clinical practitioners have further experimented with PSAD thresholds of <0.15 [ng/ml]/cm^3^ to improve its ability to diagnose HGPCa for PI-RADS ≤ 3 lesions, which is based on testing the effectiveness of PSAD in conjunction with mpMRI. Among them, 0.10 [ng/ml]/cm^3^ is one of the most studied thresholds (36, 37). In the group of patients with PI-RADS 2 lesions with pre-biopsy and AS, PSAD with 0.10 [ng/ml]/cm^3^ as a threshold was advantageous in terms of the NPV (96.2% vs. 89.7%) and leakage rate (3.8% vs. 10.3%) when compared with 0.15 [ng/ml]/cm^3^; however, this advantage was not significant in patients with PI-RADS 3 lesions (38, 39). Therefore, patients with PIRADS ≤ 2 and PSAD < 0.10 [ng/ml]/cm^3^ do not need to be biopsied, patients with PIRADS 3 and PSAD > 0.15 [ng/ml]/cm^3^ should be biopsied, and patients with HGPCa risk between these should undergo biopsy according to their wishes. This is consistent with risk reporting in the European Association of Urology Guidelines (40). Because PSAD is affected by PV (14), clinicians should calculate the PV after mpMRI and determine whether patients with PI-RADS ≤ 3 lesions can be further diagnosed definitively by PSAD. Adjusting for conditions can potentially improve the accuracy of PSAD in diagnosing HGPCa in patients with PI-RADS ≤ 3 lesions.

### 3.2 MpMRI combined with Proclarix

The combination of Proclarix and mpMRI significantly improved the accuracy of diagnosing HGPCa compared to Proclarix alone and mpMRI alone (41) (Table 1). In a cohort of patients with PI-RADS ≤ 2 lesions, Proclarix enabled 30.0% of patients to avoid biopsy while accurately detecting all patients with HGPCa missed by mpMRI. In a cohort of patients with PI-RADS 3 lesions, Proclarix enabled 21.3% of patients to avoid biopsy and detected all patients with HGPCa (43). Therefore, for patients with PI-RADS ≤ 3 lesions, the highly sensitive Proclarix is an excellent complementary test.

**Table 1:** Diagnostic performance of mpMRI combined biomarkers vs. biomarkers alone in patients with HGPCa.

Test	Intended use	Se (%)	Sp (%)	Avoid biopsy (%)	HGPCa missed (%)	AUC
PSAD (11, 14, 37, 38)	PSA ≥ 4 ng/ml Abnormal DRE PV ≤ 75 cm^3^ In AS	90.0	34.4	28.4	10.0	0.670
mpMRI + PSAD (14, 37, 38, 42)	PI-RADS 2–3 PV ≤ 75 cm^3^ In AS	87.5	60.5	58.4	6.5	0.780
Proclarix (16, 17, 41)	PSA 2–10 ng/ml Normal DRE PV ≥ 35 cm^3^	97.0	24.0	18.2	2.6	0.750
mpMRI + Proclarix (41, 43, 44)	PI-RADS ≤ 3	100.0	25.0	30.0	0.0	0.880
T2-E (45, 46)	PSA 2.5–11 ng/ml Abnormal DRE	52.6	58.3	–	–	0.670
mpMRI + T2-E (46)	PSA ≥ 4 ng/ml PI-RADS ≥ 3	–	–	–	–	0.730
MiPS (20, 47)	PSA > 3 ng/ml	92.6	33.4	42.0	–	0.772
mpMRI + MiPS (48)	PI-RADS 3	94.0	44.0	44.0	6.0	0.730
EPI (23)	PSA 2–10 ng/ml	93.0	26.1	26.0	7.0	0.700
EPI + mpMRI (49)	PSA 4–10 ng/ml Abnormal DRE EPI ≥ 15.6	–	–	43.0	4.8	–
4Kscore (27, 28)	PSA 4–10 ng/ml Abnormal DRE In AS	–	–	23.4	3.7	0.779
4Kscore + mpMRI (27, 50)	PSA 4–10 ng/ml Abnormal DRE 4Kscore ≥7.5%	–	–	34.2	2.7	0.853
SelectMDx (29, 51)	PSA ≥ 3 ng/ml (initial biopsy) PSA < 10 ng/ml (in AS)	83.0	36.8	53.5	12.9	0.684
mpMRI + SelectMDx (51)	PI-RADS ≤ 3	54.8	91.9	40.0	3.2	0.730
PHI (33, 52, 53)	PSA 4–10 ng/ml Normal DRE In AS	91.7	43.6	35.3	8.3	0.760
mpMRI + PHI (53–56)	PI-RADS 3 In AS	79.0	81.0	50.0	4.2	0.884
PHID (34, 35)	PSA ≥ 4 ng/ml Abnormal DRE PV ≤ 50 cm^3^	91.7	56.2	49.3	8.3	0.826
mpMRI + PHID (34, 57, 58)	PI-RADS 1–5 PV ≤ 50 cm^3^	94.7	70.0	35.3	7.7	0.900

mpMRI, multiparametric magnetic resonance imaging; HGPCa, high grade prostate cancer; Se, sensitivity; Sp, specificity; AUC, area under the curve; PSAD, prostate-specific antigen density; PSA, prostate specific antigen; AUC, area under the curve; DRE, digital rectal examination; PV, prostate volume; AS, active surveillance; PI-RADS, prostate imaging reporting and data system; T2-E, *TMPRSS2:ERG* gene fusion; MiPS, Michigan prostate score; EPI, ExoDX prostate intelliscore; 4Kscore, four kallikrein score; SelectMDx, select molecular diagnosis; PHI, prostate health index; PHID, prostate health index density.

### 3.3 MpMRI combined with T2-E

In a prospective study that included 158 patients, the combination of T2-E and mpMRI did not show an advantage in the diagnostic accuracy of HGPCa compared to a baseline model consisting of age, PSA, previous biopsy history, and family history (AUC: 0.730 vs. 0.740) (46). The high economic cost of this combined protocol, the lack of improvement in diagnostic accuracy, and the low incidence of T2-E gene fusion in Asian patients make the combination unsuitable for HGPCa diagnosis in Asians (19, 46).

### 3.4 MpMRI combined with MiPS

In patients with PI-RADS 3 lesions, using MiPS as the basis for biopsy decisions would allow 44.0% of patients to avoid biopsy and miss only 6.0% of cases of HGPCa (48). However, the accuracy of MiPS combined with mpMRI for diagnosing HGPCa was lower than that of MiPS alone (AUC: 0.730 vs. 0.772), which is possibly due to differences in disease severity between the patients in the two studies (20, 48). It should be noted that in the validation cohort in which Tomlins et al. developed the MiPS model, the prevalence of HGPCa was 68.0%, and the median MiPS was 50, whereas in the study evaluating mpMRI in combination with MiPS, the percentage of patients with HGPCa was 49.0% and the median MiPS was 35.4 (20, 48). When comparing the ability to diagnose HGPCa, a high prevalence of HGPCa in a patient cohort can mask MiPS misdiagnosis.

### 3.5 MpMRI combined with EPI

The EPI-mpMRI regimen with anterior biomarkers ensured that more unnecessary biopsies were avoided than the mpMRI-EPI regimen (43.0% vs. 19.3%) with a low missed diagnosis rate (4.8%) while reducing the use of mpMRI by 39.9% (49); therefore, it greatly reduces the medical burden on the patients. According to the recommendation of de la Calle et al. (49), patients with suspected PCa should first undergo an EPI examination, and if the result is <15.6, a PSA follow-up examination could be performed. For patients with 15.6 ≤ EPI < 19, the decision for a subsequent biopsy or PSA follow-up examination should be made according to the mpMRI result. If the EPI ≥ 19, then prostate biopsy should be performed after mpMRI examination.

### 3.6 MpMRI combined with 4Kscore

The 4Kscore-mpMRI protocol resulted in 39.4% of patients avoiding biopsy, a reduction in the use of mpMRI by 29.5%, and a missed diagnosis rate of only 5.6% (49, 59). According to this examination protocol, patients can first undergo a 4Kscore examination, and if the result is <7.5%, they could choose PSA follow-up examination. If 7.5% ≤4Kscore <20.0%, the decision for a subsequent biopsy or PSA follow-up examination is made based on the results of the mpMRI. Furthermore, if the result of 4Kscore is ≥20.0%, prostate biopsy is performed after the mpMRI examination. Given the ability of the 4Kscore combined with mpMRI to diagnose HGPCa and the ability of the 4Kscore to monitor disease progression (27, 28), 4Kscore can be added to the monitoring methods for patients under AS in conjunction with mpMRI for the early detection of disease progression, which is in line with the current NCCN guidelines, encouraging the use of mpMRI to monitor the condition of patients under AS (3).

### 3.7 MpMRI combined with SelectMDx

A meta-analysis (29) including data from 1,328 patients showed that SelectMDx alone and mpMRI alone had a similar accuracy in the diagnosis of HGPCa. In contrast, the combination of the two ensured a higher number of true-positive cases (527/1,000) and fewer false-negative cases (13/1,000). In recent years, researchers have devoted themselves to studying the combined strategy of SelectMDx and mpMRI (51, 60, 61). Maggi et al. (51) designed seven examination strategies for SelectMDx and mpMRI to diagnose HGPCa and found that the mpMRI-SelectMDx protocol was the optimal choice by comparison. Patients with PSA > 3 ng/ml and/or abnormal DRE were first examined by mpMRI, and prostate biopsy was performed if mpMRI was positive (PI-RADS > 3). If PI-RADS was ≤3, SelectMDx was performed, and a biopsy was performed only if SelectMDx results were abnormal. Clinicians following this protocol to guide biopsy decisions can avoid unnecessary biopsies in 40.0% of patients with a missed diagnosis rate of only 3.2% for HGPCa.

### 3.8 MpMRI combined with PHI

The ability of PHI to predict HGPCa in specimens from biopsy or radical prostatectomy is further enhanced when coupled with mpMRI (55, 62, 63). Diagnostic data on PHI-mpMRI (64) and mpMRI-PHI protocols (53, 54, 65) suggest that the mpMRI-PHI protocol is the better choice. When a PHI threshold of 27 was used, mpMRI-PHI had a sensitivity of 100.0% and an AUC of 0.884 for diagnosing HGPCa in a PI-RADS 3 population (32, 56). Thus, PHI could be used as a complementary screening tool after mpMRI to guide biopsy decisions in patients with PI-RADS 3 lesions. If mpMRI is used with PHI to rule out low-risk patients under AS, the NPV can be as high as 98.0% while enabling 20.0% of patients to avoid an unnecessary prostate biopsy (54).

### 3.9 MpMRI combined with PHID

The PHI demonstrated a similar diagnostic ability to PHID in patients with PSA 4–10 ng/ml and negative DRE (AUC: 0.760 vs. 0.770). In contrast, PHID, as a derivative of PHI, broadened the scope of use and further improved the diagnostic accuracy. In patients with PSA > 10 ng/ml, PHID was a superior diagnostic tool for HGPCa to PHI (AUC: 0.840 vs. 0.790) (52). Given that PHID is affected by the PV (34), the decision of whether to perform PHID in patients with PSA ≥ 4 ng/ml and/or negative or suspicious DRE could be based on the PV on the mpMRI report. In a group of patients with a median PV ≤ 50 cm^3^ and PSA ≥ 4 ng/ml, the AUC for PHID combined with mpMRI to diagnose HGPCa was as high as 0.900 (58).

## 4 Discussion

Among the abovementioned combined protocols, mpMRI with Proclarix, EPI, 4Kscore, PHI, and PHID performed exceptionally well in diagnosing HGPCa, with all demonstrating a high accuracy (AUC > 0.850). Combination regimens that use anterior biomarkers, such as EPI-mpMRI and 4Kscore-mpMRI, can help to avoid unnecessary prostate biopsies and reduce the use of mpMRI, conducive to reducing the healthcare burden on the patients. If the mpMRI results are negative or suspicious, elevated PSA persists, and PCa is highly suspected, clinicians could choose to assess the Proclarix, PHI, or PHID for further clarifications and avoid prostate biopsy if subsequent biomarker tests are negative. Considering the ability to diagnose HGPCa and the frequency of T2-E fusion genes, mpMRI combined with T2-E was found to not be suitable for the diagnosis of HGPCa in Asians.

Germline mutations, DNA methylation, molecular alterations of key PCa genes, genome-wide expression profiles, and epidemiologic characteristics differ significantly among patients with PCa of different races due to genetic factors, living environment, and medical care (66, 67). The incidence of PCa in European Americans is 68 cases per 100,000 men, with a 10-year survival rate of 86.0%, while the incidence of PCa in Asians ranges from 2 to 10 cases per 100,000 men, with a 10-year survival rate of 36.2%. Genetically, the three most common mutated genes and mutation frequencies are *BRCA2* (2.6%), *BRCA1* (1.3%), and *HOXB13* (1.3%) in African Americans, and *BRCA2* (4.1%), *BRCA1* (2.7%), and *ATM* (2.7%) in Asians, with mutations in *BRCA2* associated with a poor prognosis (67–70). The incidence of PCa in Asia is lower than in Europe and North America; however, the prognosis is poor. This inspires us not to neglect the early and accurate diagnosis of Asian patients with PCa, and there is a need to explore the applicability of diagnostic protocols in Asian populations. Although fewer clinical trials on mpMRI combined with biomarkers have been conducted in Asian populations, limited data suggest that such combination protocols have good diagnostic accuracy in Asian populations (Table 2).

**Table 2:** MpMRI combined with biomarkers for the diagnosis of HGPCa in the Asian population.

Test	Country	Study type	Sample size	Avoid biopsy (%)	HGPCa missed (%)	AUC
mpMRI + PSAD (71)	China	Retrospective	*N* = 240	46.3	0.0	0.786
mpMRI + PHI (72)	Korea	Retrospective	*N* = 232	–	–	0.881
mpMRI + PHI (55)	China	Retrospective	*N* = 315	–	–	0.850
mpMRI + PHI (53)	China	Prospective	*N* = 102	50.0	4.2	0.873
mpMRI + PHI (56)	China	Prospective	*N* = 164	69.1	5.9	0.884
mpMRI + PHID (73)	Korea	Retrospective	*N* = 521	45.9	0.0	0.884
mpMRI + PHID (57)	China	Retrospective	*N* = 128	–	–	0.913
mpMRI + PHID (74)	China	Prospective	*N* = 89	94.44	15.0	0.829
mpMRI + PHID (75)	China, Singapore	Retrospective	*N* = 1,215	–	–	0.850

mpMRI, multiparametric magnetic resonance imaging; HGPCa, high-grade prostate cancer; AUC, area under the curve; PSAD, prostate-specific antigen density; PHI, prostate health index; PHID, prostate health index density.

Theoretically, biomarkers that can recognize HGPCa can also be used to monitor disease progress in patients under AS. In the future, the accuracy of combined mpMRI and biomarkers in the diagnosis of HGPCa could be evaluated in prospective, multicenter cohorts of patients under AS. The combined protocol could be extended from pre-biopsy patients to patients under AS to provide them with additional monitoring options. The accuracy of mpMRI combined with biomarkers for the diagnosis of HGPCa in Asians needs to be further evaluated in large-sample, prospective clinical trials.

## 5 Conclusion

MpMRI-Proclarix, mpMRI-PHI, mpMRI-PHID, 4Kscore-mpMRI, and EPI-mpMRI were shown to improve the diagnostic accuracy of HGPCa, enabling patients to avoid unnecessary biopsy or mpMRI. Moreover, mpMRI combined with biomarkers to diagnose HGPCa is feasible in Asians.
